# Aggressive high-grade NF2 mutant meningiomas downregulate oncogenic YAP signaling via the upregulation of VGLL4 and FAT3/4

**DOI:** 10.1093/noajnl/vdae148

**Published:** 2024-08-24

**Authors:** Abigail G Parrish, Sonali Arora, H Nayanga Thirimanne, Dmytro Rudoy, Sebastian Schmid, Philipp Sievers, Felix Sahm, Eric C Holland, Frank Szulzewsky

**Affiliations:** Human Biology Division, Fred Hutchinson Cancer Center, Seattle, Washington, USA; Human Biology Division, Fred Hutchinson Cancer Center, Seattle, Washington, USA; Human Biology Division, Fred Hutchinson Cancer Center, Seattle, Washington, USA; Human Biology Division, Fred Hutchinson Cancer Center, Seattle, Washington, USA; Human Biology Division, Fred Hutchinson Cancer Center, Seattle, Washington, USA; Clinical Cooperation Unit Neuropathology, German Consortium for Translational Cancer Research (DKTK), German Cancer Research Center (DKFZ), Heidelberg, Germany; Department of Neuropathology, Institute of Pathology, University Hospital Heidelberg, Heidelberg, Germany; Hopp Children’s Cancer Center Heidelberg (KiTZ), Heidelberg, Germany; Clinical Cooperation Unit Neuropathology, German Consortium for Translational Cancer Research (DKTK), German Cancer Research Center (DKFZ), Heidelberg, Germany; Department of Neuropathology, Institute of Pathology, University Hospital Heidelberg, Heidelberg, Germany; Seattle Translational Tumor Research Center, Fred Hutchinson Cancer Center, Seattle, Washington, USA; Human Biology Division, Fred Hutchinson Cancer Center, Seattle, Washington, USA; Department of Neurosurgery, Clinical Neurosciences Center, University of Utah, Salt Lake City, Utah, USA; Huntsman Cancer Institute, University of Utah Health Sciences Center, Salt Lake City, Utah, USA; Human Biology Division, Fred Hutchinson Cancer Center, Seattle, Washington, USA

**Keywords:** hippo, meningioma, NF2, VGLL4, YAP1

## Abstract

**Background:**

Meningiomas are the most common primary central nervous system tumors in adults. Although generally benign, a subset is of higher grade and ultimately fatal. Around half of all meningiomas harbor inactivating mutations in NF2, leading to deregulation of oncogenic YAP1 activity. While benign NF2 mutant meningiomas exhibit few genetic events in addition to NF2 inactivation, aggressive high-grade NF2 mutant meningiomas frequently harbor a highly aberrant genome. It is unclear if NF2 mutant meningiomas of different grades are equally reliant on YAP activity.

**Methods:**

We analyzed bulk and single-cell RNA-Seq data from a large cohort of human meningiomas for the expression of YAP1 target genes and Hippo effectors as well as in vitro cell line experiments.

**Results:**

Aggressive NF2 mutant meningiomas harbor decreased expression levels of YAP1 target genes and increased expression levels of the YAP1 antagonist VGLL4 and the upstream regulators FAT3/4 compared to their benign counterparts. Decreased expression of YAP1 target genes as well as high expression of VGLL4 and FAT3/4 is significantly associated with an increased risk of recurrence. In vitro, overexpression of VGLL4 resulted in the downregulation of YAP activity in benign NF2 mutant meningioma cells, confirming the direct link between VGLL4 expression and decreased levels of YAP activity observed in aggressive NF2 mutant meningiomas.

**Conclusions:**

Our results shed new insight into the biology of benign and aggressive NF2 mutant meningiomas and may have important implications for the efficacy of therapies targeting oncogenic YAP1 activity in NF2 mutant meningiomas.

## Graphical Abstract

**Graphical Abstract:**
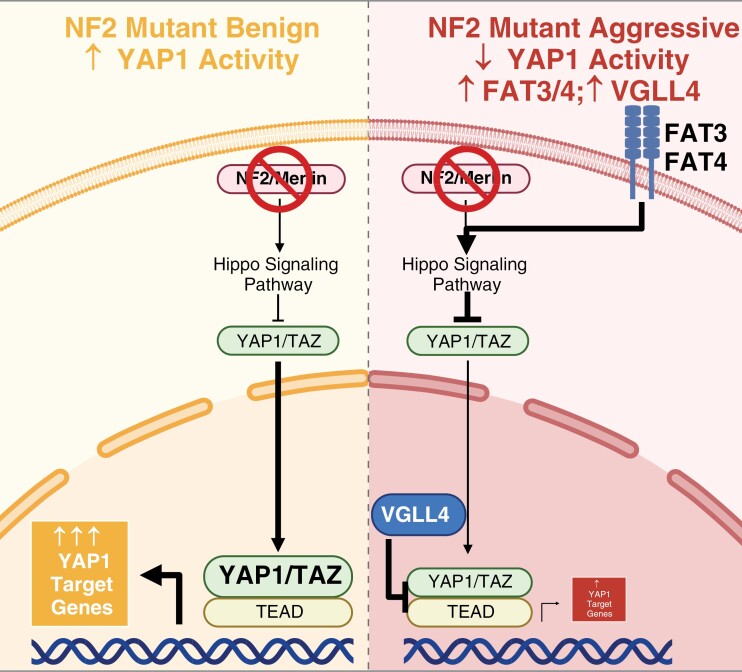

**Key Points**

- Aggressive NF2 mutant meningiomas downregulate YAP activity.
- This is achieved in part by the up-regulation of VGLL4 or FAT3/4.
- Expression of VGLL4 in benign NF2 mutant meningioma cells leads to a downregulation of YAP activity.

**Importance of the Study**

Around half of meningiomas harbor inactivating mutations in the NF2 gene, leading to the deregulation of oncogenic YAP activity in these tumors. Since benign NF2 mutant meningiomas harbor few mutations in addition to functional NF2 loss it is conceivable that deregulated YAP signaling is the main driver in these tumors and a potential therapeutic target for YAP1-TEAD inhibitors. By contrast, aggressive high-grade NF2 mutant meningiomas harbor a highly aberrant genome with several chromosomal gains and losses in addition to NF2 loss, leading to the activation of additional mitogenic pathways. Our results suggest that aggressive NF2 mutant meningiomas downregulate YAP activity and harbor lower expression levels of several YAP target genes, in part by the upregulation of the YAP1 antagonist VGLL4 and the upstream regulators FAT3/4. These results may have important implications for the efficacy of YAP1-TEAD inhibitors in benign versus aggressive NF2 mutant meningiomas.

Meningiomas are the most common primary central nervous system (CNS) tumors in adults.^1^ Although only a subset of meningiomas shows aggressive growth behavior, atypical and anaplastic meningiomas frequently invade into the brain and tend to recur even after multiple rounds of surgery, chemo-, and radiation therapy. Around half of all meningiomas exhibit functional loss of the *NF2* gene, either due to loss of chromosome 22, inactivating point mutations, or gene fusions. Aggressive meningiomas occur in all molecular subgroups but are enriched in NF2 mutant meningiomas. While benign NF2 mutant meningiomas usually only exhibit functional NF2 loss and rarely harbor additional recurrent mutations or chromosomal aberrations, aggressive NF2 mutant meningiomas generally harbor a more aberrant genome with several recurrent chromosomal gains and losses in addition to functional NF2 inactivation/chromosome 22 loss, such as losses of chromosomes 1p, 4, 6, 10, and 14.^2,3^

NF2 (encoding for the protein Merlin) is a potent tumor suppressor that functions as an upstream regulator of the Hippo Signaling pathway that translates mechanical stimuli into transcriptional signals.^4,5^ NF2, in a complex with Expanded (FRMD6) and Kibra (WWC1), activates a core cascade of serine-threonine kinases (STK3/4, LATS1/2) that ultimately phosphorylate the transcriptional coactivator YAP1 (Yes-associated protein; and its paralog TAZ (transcriptional coactivator with PDZ-binding motif)) and inhibits its functions.^6^ Several studies have detected increased YAP activity in NF2 mutant meningioma tumors and/or cell lines^7,8^ and recurrent YAP1 gene fusions have been identified in a subset of pediatric NF2 wild-type meningiomas^9,10^ and other CNS and non-CNS tumors.^4,11^ In addition to the canonical Hippo Signaling pathway working through NF2, gigantic cadherin proteins FAT and Dachsous positively regulate Hippo signaling and inhibit Yorkie (the YAP1 ortholog in Drosophila).

YAP1 is a transcriptional coactivator that predominantly functions through its interaction with the family of TEAD (transcriptional enhanced associate domain) transcription factors. Ablation of the YAP1-TEAD interaction, either through mutagenesis or pharmacological inhibition, results in functional YAP inactivation.^12^ Similarly, pharmacological inhibition of the YAP1-TEAD interaction with small molecule inhibitors can inhibit the expression of YAP1 downstream targets (such as CYR61/CCN1 and CTGF/CCN2) as well as the growth of YAP1-driven cells. Several of these inhibitors are currently under investigation in clinical trials.^13^ TEAD transcription factors can also interact and bind to other cofactors, such as VGLL proteins. Vestigial-like family members 1–4 (VGLL1-4) are transcriptional cofactors that compete with YAP1 for TEAD binding.^14–16^

Historically, meningiomas have been classified into 3 CNS WHO grades, based on established histologic criteria. However, the histological grade frequently does not accurately predict tumor growth, behavior, and recurrence, highlighting the need for additional classifications based on genetic, epigenetic, and transcriptional markers. Recent large-scale sequencing efforts have utilized several approaches, including DNA methylation-based sequencing, whole exome sequencing, and bulk RNA sequencing to classify meningiomas beyond the classic CNS WHO grading system.^2,3,17,18^ To gain a deeper insight into which pathways are activated in different meningioma subclasses and grades, we have recently collected RNA Seq data of 1298 human meningiomas and performed UMAP analysis to identify clusters that correlate with clinically relevant subgroups. We observed that RNA Seq analysis was able to predict tumor grade, recurrence, and functional NF2 status, and observed significant overlaps with clusters derived from DNA methylation-based and whole exome sequencing, suggesting that RNA Seq-based classification yields compatible results.^19^

Several open questions remain about the pathobiology of both benign and aggressive NF2 mutant meningiomas. In this study, we utilize our collection of RNA-Seq data from 1298 human meningioma samples as well as additional human meningioma single-cell sequencing data to investigate the levels of YAP1 activity in the different meningioma subclusters. We find that aggressive NF2 mutant meningiomas exhibit decreased levels of YAP1 activity, the putative oncogenic driver in benign NF2 mutant meningiomas. We then identify elevated expression of the YAP1 antagonist VGLL4 in a subset of aggressive NF2 mutant meningiomas as one possible route of functional YAP1 inhibition. Lastly, using an in vitro cell culture model, we show that overexpression of VGLL4 in benign NF2 mutant meningioma cells leads to the downregulation of canonical YAP1 target genes.

## Material and Methods

### RNA Sequencing Data of Human Meningiomas

The collection of bulk RNA Seq data from human meningiomas has previously been published.^19^ Single-cell RNA Seq data of human meningioma tumors has previously been published,^20^ was obtained from the data repository of the Department of Neuropathology at the University Hospital Heidelberg, and is available upon request. RNA-Seq data from human Ben-Men-1 cells expressing either VGLL4 or GFP can be accessed at the GEO database at GSE263122. The code used to process and analyze the data is available at https://github.com/sonali-bioc/SzulzewskyVGLL4Paper.

### Bulk RNA-Seq Analysis

For analysis of human meningioma tumors, Bulk RNASeq data (vst counts), UMAP coordinates, and metadata for 1298 human Meningioma tumors from^19^ were downloaded. CNS WHO grade was based on the 2016 edition of the CNS tumor classification.^21^ R package DESeq2 was used to find differentially expressed genes (DEGs) between Cluster A (NF2 mutant aggressive) vs Cluster B (NF2 mutant benign). A threshold of fold change of 1.5 (or logFC of 0.58) and a *P*-adjusted value <.05 were used to determine significantly regulated DEGs.

For analysis of RNA-Seq data from Ben-Men-1 cells expressing either VGLL4 or GFP, Raw sequencing reads were checked for quality using FastQC (https://www.bioinformatics.babraham.ac.uk/projects/fastqc/). RNA-seq reads were aligned to the hg38 assembly using gencode v39 and STAR2^22^ and counted for gene associations against the UCSC genes database with HTSeq.^23^ Differential Expression analysis for RNASeq Data was performed using R/Bioconductor package DESeq2 and edgeR. A log2fold change cutoff of 1 (fold change of 2) and FDR <0.05 were used to find transcriptionally regulated genes.

### Survival Analysis

For each gene, using the gene expression for samples present in clusters A and B, the patient samples were divided into 2 groups—group 1 contained samples whose gene expression was less than first quantile and group 2 contained samples whose gene expression was higher than third quantile of all samples. R package survival (https://cran.r-project.org/web/packages/survival/index.html) and survminer (https://cran.r-project.org/web/packages/survminer/index.html), were then used to draw a KM curve and find survival differences between the two groups.

### Single-Cell RNA-Seq Data Analysis

Seurat objects were constructed for 26 single-cell RNA-Seq samples of human meningiomas.^24^ Cells from the 26 single-cell RNA-Seq samples were further filtered to remove immune cells based on 49 marker genes (Supplementary Table 1) spanning a variety of immune cell types. Next SCTransform from Seurat was used to process the data, followed by building a UMAP and forming clusters. Metadata extracted for the remainder cells was used to divide cells into different groups for CNS WHO grade^21^ and methylation cluster (MC) status. Dotplots and violin plots for key genes were made using the function DotPlot() and VlnPlot() respectively from Seurat. FindMarkers() from Seurat was used to find differentially expressed genes between the different MC status (ben-1 vs ben-2, ben-1 vs int-A, ben-1 vs int-B, ben-1 vs mal). R package, R function, DoHeatmap() were used to make heatmaps. All analysis was done using R 4.3.3 and Seurat (v 5.0.0).

### Enrichment Analysis and Data Visualization

Gene set enrichment analysis was performed against the MsigDB database with the KEGG gene sets using enrichR^25^ package. The resulting enriched genesets and pathways were filtered via a threshold of FDR <0.05. Fisher hypergeometric tests were implemented in R using the function phyper() to see if genes in one set were over-represented, compared to other gene sets. R package pheatmap (https://cran.r-project.org/web/packages/pheatmap/index.html) was used to make heatmaps. Volcano plots were made using R (v4.3.3). All other plots were made using ggplot2.

### Plasmid Preparation

Primers used for plasmid generation are listed in Supplementary Table 2A. Additional plasmids used in this study are listed in Supplementary Table 2B.

### Luciferase Assays

HEK293 cells were cultured in DMEM, 10% FBS, and 1% Penicillin/Streptomycin. If not indicated differently, HEK293 cells were seeded into white 96-well plates at 10 000 cells/well the day prior to transfection. Cells were then transfected with the indicated plasmids and a plasmid containing Renilla using Lipofectamin 3000 (Thermo Fisher Scientific) according to the manufacturer’s instructions. Luciferase activity was measured 24 hours after transfection using the Dual-Glo Luciferase Assay System (Promega) on a Veritas Microplate Luminometer.

### Lentiviral Transductions

For virus production, pLJM1 (Addgene) constructs containing the inserts of interest were transfected into 293T cells, along with psPAX and pMD2.G packaging plasmids (Addgene), using polyethylenimine (Polysciences). Fresh media was added 24 hours later, and the viral supernatant was collected and filtered through a 0.45 µm filter 24 hours later. For infection of NIH3T3 or Ben-Men-1 cells, 1 × 10^5^ cells/well were seeded into 6-well plates. Lentivirus was used unconcentrated and cells were infected at a MOI <1 24 hours after seeding. Seventy-two hours after seeding, selection was begun for cells successfully expressing the constructs using 1 μg/mL puromycin (for 3 days).

### Live-Cell Imaging

Ben-Men-1 cells were purchased through DSMZ (ACC 599) and were cultured with DMEM with 20% FBS and 1% Penicillin-Streptomycin. Ben-Men-1 cells (expressing either GFP or VGLL4) were seeded at 500 cells per well in tissue culture-treated 96-well plates. 2D growth of cells was monitored using live cell imaging every 24 hours for 108 hours in the Incucyte ZOOM system (Essen). Average phase object confluency was used for analysis. Viability was measured using the CellTiter-Glo 2.0 Cell Viability Assay (Promega G9241) according to the manufacturer’s instructions. Luminescence was detected using a Veritas Microplate Luminometer.

### RNA Isolation, PCR, and RNA Sequencing

RNA was extracted using the Qiagen RNeasy Mini Kit according to the manufacturer’s instructions. Genomic DNA was removed by on-column DNase digestion. Total RNA integrity was checked using an Agilent 4200 TapeStation (Agilent Technologies, Inc.) and quantified using a Trinean DropSense96 spectrophotometer (Caliper Life Sciences). RNA-seq libraries were prepared from total RNA using the TruSeq Stranded mRNA kit (Illumina, Inc.). Library size distribution was validated using an Agilent 4200 TapeStation (Agilent Technologies). Additional library QC, blending of pooled indexed libraries, and cluster optimization was performed using Life Technologies’ Invitrogen Qubit 2.0 Fluorometer (Life Technologies-Invitrogen). RNA-seq libraries were pooled (70-plex) and clustered onto a P3 flow cell. Sequencing was performed using an Illumina NextSeq 2000 employing a paired-end, 50 base read length (PE50) sequencing strategy. For quantitative real-time PCR, RNA was transcribed into cDNA using the SuperScript III kit. PCR experiments were carried out on a QuantStudio 7 Flex Real-Time PCR System. For primer sequences see Supplementary Table 2A.

### Ethics Statement

Studies were conducted in accordance with the US Common Rule ethical guidelines.

## Results

### Aggressive NF2 Mutant Meningiomas Harbor Reduced Levels of YAP Activity Compared to Benign NF2 Mutant Meningiomas

We have previously collected a large cohort of 1298 bulk RNA-Seq samples of human Meningiomas and have applied Uniform Manifold Approximation and Projection (UMAP), a dimensionality reduction method, on batch-corrected, normalized transcript counts to create a reference UMAP. DBSCAN (density-based spatial clustering of applications with noise) was then used to delineate regional distinct clusters that corresponded with metadata (such as time to recurrence, CNS WHO grade) and were able to separate NF2 mutant meningiomas into a benign and an aggressive cluster.

**Figure 1.**
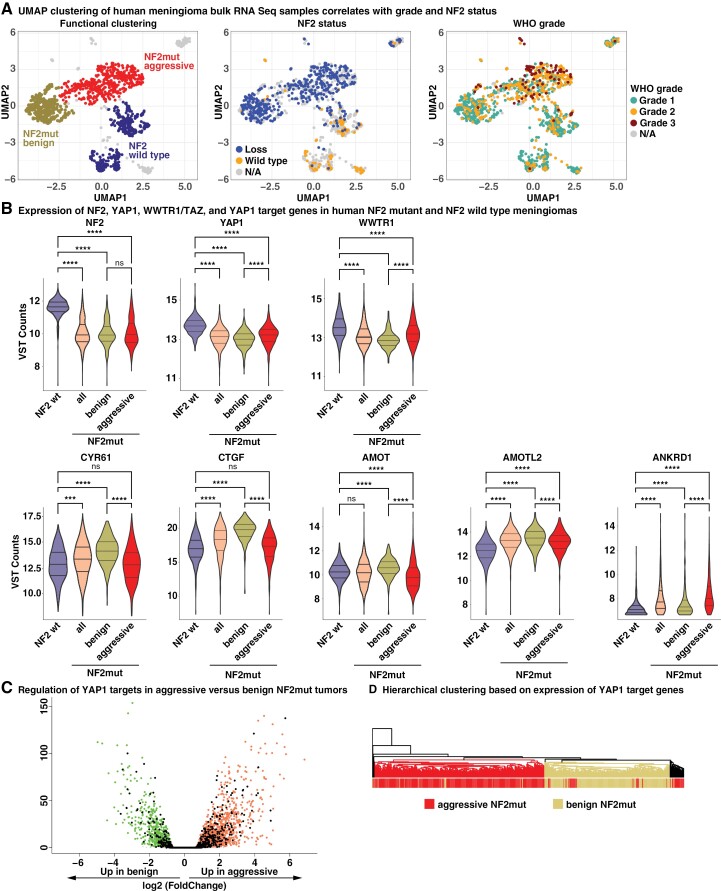
Aggressive NF2 mutant meningiomas display decreased levels of YAP activity. (A) Reference UMAPs showing clustering of human meningiomas based on bulk RNA-Seq data. Samples are colored by cluster (benign NF2 mutant, aggressive NF2 mutant, NF2 wild type), NF2 status, or WHO grade. (B) Expression of Hippo pathway genes and YAP1 target genes in bulk RNA-Seq data of different clusters of human meningiomas. (C) Expression of YAP target genes (black dots) in benign versus aggressive human NF2 mutant meningiomas. (D) Hierarchical clustering of human meningioma bulk RNA-Seq samples based on the expression of 2212 YAP target genes is able to separate benign and aggressive NF2 mutant meningiomas. Statistical analysis was done with One-way ANOVA (B). (***) *P* ≤ .001; (****) *P* ≤ .0001.

To explore the levels of YAP activity in different subtypes of meningioma (NF2 wild type, benign, and aggressive NF2 mutant), we analyzed the expression of NF2, YAP1, and the YAP1 paralogue TAZ/WWTR1 in our cohort of 1298 bulk RNA-Seq samples from human meningiomas, including both NF2 mutant and wild type tumors. We subdivided NF2 mutant meningiomas into benign (*n* = 290) and aggressive (*n* = 476) tumors based on the previously established clusters (Figure 1A, Supplementary Figure 1A, B).^19^ Since the activity of YAP1 is largely regulated at the protein level and not at RNA level, we also investigated the expression of several YAP1 target genes (CYR61/CCN1, CTGF/CCN2, AMOT, AMOTL2, and ANKRD1).

The expression of NF2, YAP1, and TAZ were all significantly decreased in NF2 mutant compared to NF2 wild-type tumors. By contrast, the expression of the YAP1 target genes CTGF, CYR61, AMOTL2, and ANKRD1 was significantly higher in NF2 mutant tumors compared to NF2 wild-type meningiomas (Figure 1B).

When comparing aggressive to benign NF2 mutant tumors, we detected no significant difference in the expression of NF2 between benign and aggressive NF2 mutant meningiomas, whereas the expression of YAP1 and TAZ was significantly higher in aggressive NF2 mutant tumors. Since the activity of YAP1 and TAZ is primarily regulated on protein level, we then again analyzed the expression of several YAP1 target genes as a surrogate for actual YAP activity. By contrast, we detected significantly decreased expression levels of the YAP1 target genes CTGF (3.2-fold), CYR61 (2-fold), AMOT (1.7-fold), and AMOTL2 (1.5-fold) in aggressive compared to benign NF2 mutant meningiomas (Figure 1B, Supplementary Figure 1C). Of note, the YAP1 target gene ANKRD1 was expressed at significantly higher levels in aggressive versus benign NF2 mutant tumors (1.8-fold).

To analyze the differences in YAP activity between benign versus aggressive meningiomas on a broader scale, we calculated the differentially-expressed genes between benign and aggressive meningiomas (Fold change ≥1.5, *P*-adjusted ≤.05). We observed 1836 upregulated and 843 downregulated genes in aggressive NF2 mutant meningiomas (compared to benign NF2 mutant tumors). We compared these DEGs to a dataset of YAP1 target genes^26^ and observed that 17.3 percent (382 out of 2212 genes) of YAP1 target genes were significantly up- or downregulated between benign and aggressive NF2 mutant meningiomas (*P* = 5.34 × 10^−27^; Figure 1C). Unsupervised hierarchical clustering based on the expression of these 2212 YAP1 target genes was also able to separate benign from aggressive NF2 mutant meningiomas (Figure 1D).

Taken together, our data suggests that aggressive NF2 mutant meningiomas harbor different levels of YAP activity and downregulate several YAP target genes compared to benign NF2 mutant meningiomas.

### Decreased YAP Activity in Aggressive Meningiomas is Also Observed at the Single-Cell Level

Since benign NF2 mutant meningiomas exhibit increased immune cell infiltration compared to their aggressive counterparts,^20^ we used single-cell RNA sequencing data from 26 human meningiomas to assess the expression of YAP1 target genes in the tumor cells proper at the single-cell level^20^ (Supplementary Table 1A). Samples were classified based on the Heidelberg methylation classifier^3^ into either ben-1 (7 samples), ben-2 (3 samples), int-A (5 samples), int-B (2 samples), or mal (9 samples) subtypes. We excluded immune cells based on the expression of 49 marker genes (Supplementary Table 1B).

**Figure 2.**
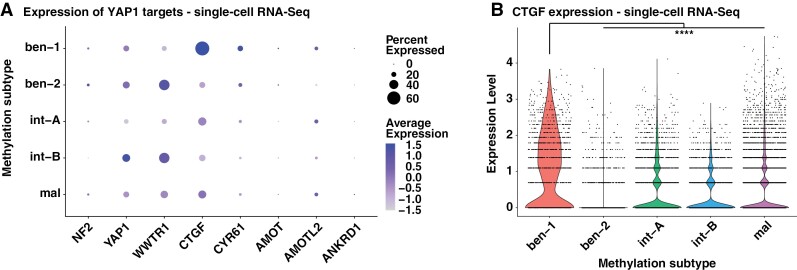
Expression of YAP target genes is decreased in aggressive type (int-A, -B, mal) human meningioma tumor cells on a single-cell level. (A) Expression of Hippo Pathway effectors and YAP1 target genes in single-cell RNA-Seq data of human meningioma tumor cells of different methylation subtype tumor samples. (B) Expression of the YAP1 target gene CTGF in single-cell RNA-Seq data of human meningioma tumor cells of different methylation subtype tumor samples. Statistical analysis was done with One-way ANOVA (B). (****) *P* ≤ .0001.

As observed in the bulk RNA-Seq samples from human meningiomas, we detected significantly decreased expression levels of several YAP1 target genes (CTGF, CYR61, ANXA3) in ben-1 NF2 mutant tumor cells compared to more aggressive NF2 mutant tumor cells, confirming that the downregulation of YAP activity in aggressive meningiomas observed in bulk RNA sequencing samples is intrinsic to the tumor cells and not caused by contamination with microenvironment and immune cells (Figure 2A, B). Both CTGF and CYR61 were significantly downregulated in tumor cells of all the higher-grade/aggressive subtypes (NF2 mutant mal subtype (CTGF: 2.48-fold decrease, *P*adj = 1 × 10^−115^; CYR61: 3.32-fold decrease, *P*adj = 4.5 × 10^−41^), int-B subtype (CTGF: 6.59-fold decrease, *P*adj = 2.9 × 10^−162^; CYR61: 4.63-fold decrease, padj = 5.15 × 10^−40^), and int-A (CTGF: 3.18-fold decrease, *P*adj = 1 × 10^−85^; CYR61: 2.77-fold decrease, *P*adj = 1.33 × 10^−18^)) compared to samples of the ben-1 subtype.

Taken together, our results suggest that the decreased YAP activity in aggressive NF2 mutant meningiomas observed in bulk RNA sequencing data is also found in single-cell sequencing data and intrinsic to the tumor cells.

### Low Expression of YAP1 Targets in NF2 Mutant Meningiomas Is Associated With Shorter Time to Recurrence

**Figure 3.**
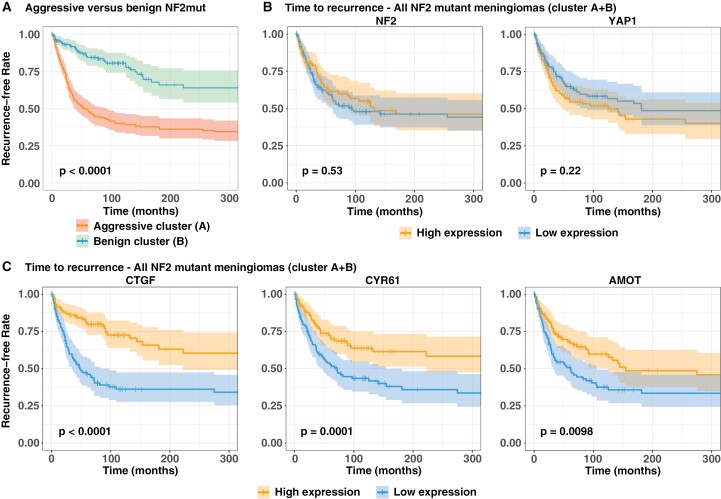
Low expression of YAP1 target genes is associated with a shorter time to recurrence in NF2 mutant meningioma. (A) Time to recurrence in aggressive versus benign NF2 mutant meningiomas. (B-C) Time to recurrence of NF2 mutant human meningiomas harboring low or high expression of either NF2 and YAP1 (B) or the YAP targets CTGF, CYR61, and AMOT (C). Statistical analysis was done with Log-rank (Mantel-Cox) test (B, C).

NF2 mutant meningiomas located in the benign and aggressive UMAP clusters are associated with a significantly different risk of recurrence (Figure 3A). We then assessed if the level of YAP activity in NF2 mutant meningiomas is significantly associated with time to recurrence. To this end, we combined all NF2 mutant meningiomas into one cohort (combining tumors of the aggressive and benign clusters), resulting in 464 NF2 mutant tumors with time-to-recurrence data. We then divided these 464 tumors into three groups of equal sample numbers based on their expression levels of a specific gene (low expression, medium expression, high expression) and compared the time to recurrence for tumors in the low expression group versus the high expression group.

We did not observe a difference in the time to recurrence for NF2 mutant meningiomas that displayed high or low expression levels of either YAP1 or NF2 (Figure 3B). By contrast, NF2 mutant meningiomas that expressed lower levels of CTGF, CYR61, or AMOT exhibited significantly shorter times to recurrence (Figure 3C). Similarly, NF2 mutant meningiomas that expressed lower levels of either CTGF, CYR61, or AMOT were enriched for tumors of higher WHO grades (Supplementary Figure 2A).

Taken together, NF2 mutant meningiomas that express lower levels of the YAP1 targets CTGF, CYR61, or AMOT are significantly associated with shorter times to recurrence and higher WHO grade.

### Aggressive NF2 Mutant Meningiomas Upregulate the YAP1 Antagonist VGLL4 As Well As the Upstream Inhibitors FAT3/4

**Figure 4.**
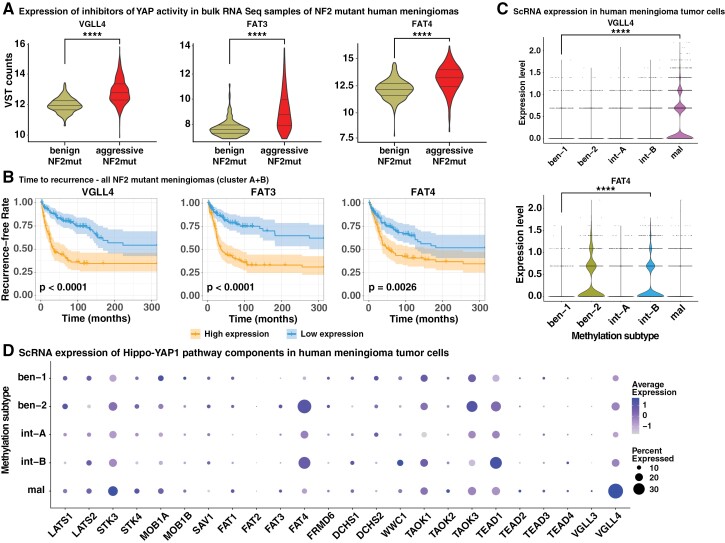
Aggressive NF2 mutant meningiomas upregulate the expression of VGLL4, FAT3, and FAT4. (A) Expression of VGLL4, FAT3, and FAT4 in bulk RNA-Seq data of benign and aggressive NF2 mutant human meningiomas. (B) Time to recurrence of NF2 mutant human meningiomas harboring low or high expression of either VGLL4, FAT3, or FAT4. (C-D) Expression of VGLL4 and FAT4 (C) and other Hippo Pathway members (D) in single-cell RNA-Seq data of human meningioma tumor cells of different methylation subtype tumor samples. Statistical analysis was done with One-way ANOVA (B) or Log-rank (Mantel-Cox) test (A, C). (****) *P* ≤ .0001.

To determine how aggressive NF2 mutant meningiomas downregulate YAP activity compared to their benign counterparts, we analyzed the expression of several regulators and mediators of YAP signaling. We analyzed the expression of different Hippo pathway upstream inhibitors of YAP1 (LATS1/2, STK3/4, MOB1A/B, SAV1, FAT1-4, FRMD6, DCHS1/2, WWC1 (KIBRA), TAOK1-3), TEAD transcription factors (TEAD1-4), as well as YAP1 competitors (VGLL1-4) in our cohort of 1298 bulk RNA-Seq samples from human meningiomas. We observed higher expression levels of several Hippo pathway members in benign NF2 mutant meningiomas, which can likely be attributed to feedback mechanisms (Supplementary Figure 3A). By contrast, we observed significantly higher expression levels of VGLL4 (2.14-fold), as well as FAT3 (10.56-fold) and FAT4 (2.27-fold) in aggressive compared to benign NF2 mutant meningiomas (Figure 4A, Supplementary Figure 3B, C). High expression of each of these three genes was associated with a significantly shorter time to recurrence in NF2 mutant tumors (Figure 4B). Importantly, high expression of both VGLL4 and FAT3 was significantly associated with a shorter time to recurrence, notably within the aggressive NF2 mutant cluster itself (Supplementary Figure 3D), suggesting that these two genes are expressed at higher levels in an especially aggressive subset of tumors.

We reutilized our single-cell RNA sequencing data and observed increased expression levels of VGLL4 and FAT3-4 in aggressive meningioma cells. Of note, we observed significant differences in the expression of VGLL4 and FAT3-4 between different methylation-classifier subgroups. In mal subtype tumor cells (compared to ben-1 subtype tumor cells) we observed a significantly increased expression of both VGLL4 (2.75-fold increase, *P*adj = 4.04 × 10^−56^) as well as VGLL3, albeit at a lower absolute level (6-fold increase, *P*adj = .004), compared to ben-1 tumor cells. Mal subtype cells also showed a significantly increased expression of both FAT3 (4.23-fold increase, *P*adj = 1.13 × 10^−6^) and to a lesser degree of FAT4 (1.58-fold increase, *P*adj = .018). By contrast, tumor cells of the int-B subtype did not show a significantly increased expression of VGLL4, VGLL3, or FAT3, but showed increased expression of FAT4 (4.31-fold increase, padj = 1.68 × 10^−55^) as well as WWC1 (1.99-fold increase, *P*adj = .0002). Int-A subtype tumor cells, similar to int-B tumor cells, showed significantly increased expression levels of FAT4 (2.53-fold increase, *P* = 2.87 × 10^−17^).

These data suggests that aggressive NF2 mutant tumors exhibit increased expression of the YAP1 antagonist VGLL4, as well as the Hippo Pathway upstream regulators FAT3 and FAT4. The mechanisms of how YAP activity is inhibited may be specific to distinct meningioma subtypes.

### Expression of the YAP1 Antagonist VGLL4 Leads to the Inhibition of YAP Activity in Benign NF2 Mutant Meningioma Cells

**Figure 5.**
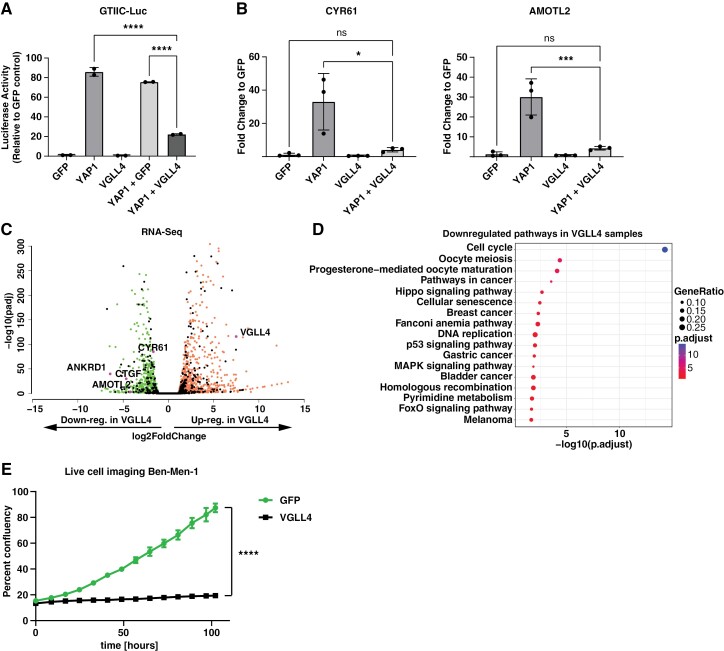
Expression of VGLL4 leads to the suppression of YAP target genes in vitro. (A) The ability of VGLL4 to suppress YAP activity in the 8xGTIIC-Luc luciferase assay. (B) Expression of the YAP1 target genes CYR61 and AMOTL2 in HEK cells upon transient transfection of either GFP, 2SA-YAP1, VGLL4, or 2SA-YAP1 + VGLL4. (C) Overexpression of VGLL4 leads to the downregulation of YAP1 target genes in human benign NF2 mutant meningioma cells (Ben-Men-1). (D) KEGG enrichment analysis of downregulated differentially expressed genes in VGLL4-expressing Ben-Men-1 cells (compared to GFP-expressing cells). (E) Live cell imaging data tracking the growth of Ben-Men-1 cells expressing either GFP or VGLL4. Statistical analysis was done with One-way ANOVA (A, B) or two-way ANOVA (E). (*) *P* ≤ .05; (***) *P* ≤ .001; (****) *P* ≤ .0001.

Since we detected increased VGLL4 expression in aggressive NF2 mutant meningioma cells, we asked what effect the overexpression of VGLL4 would have on YAP activity. To this end, we first employed a luciferase-based approach to measure the YAP activity in HEK293 cells upon VGLL4 overexpression. We transfected HEK293 cells grown at sub-confluent cell densities with the YAP1 reporter plasmid (8xGTIIC-Luc), and either GFP (control), S127/397A-YAP1 (2SA-YAP1), VGLL4, as well as a combination of 2SA-YAP1 plus either GFP or VGLL4. Overexpression of 2SA-YAP1 alone led to a significantly increased reporter activity (85.9-fold increase to GFP, *P* < .0001), whereas overexpression of VGLL4 alone resulted in significantly reduced reporter activity (0.52-fold change to GFP, *P* = .017) (Figure 5A). Furthermore, while co-expression of 2SA-YAP1 and GFP only led to a small decrease in reporter activity compared to 2SA-YAP1 alone, co-expression of VGLL4 led to a dramatic decrease in reporter activity (22.2-fold change compared to GFP). Similarly, HEK cells co-expressing 2SA-YAP1 and VGLL4 experienced significantly reduced expression levels of YAP1 target genes (CYR61, ANKRD1, AMOTL2) compared to cells expressing only 2SA-YAP1 (Figure 5B, Supplementary Figure 4A).

To explore the effects of VGLL4 overexpression in benign NF2 mutant meningioma cells, we lentivirally transduced Ben-Men-1 meningioma cells to express either GFP or VGLL4 and performed live cell imaging as well as RNA Seq analysis. VGLL4 overexpression led to the significant deregulation of a large number of YAP1 target genes (297 out of 2212, *P* = 6.8 × 10^−32^), including the downregulation of the pivotal YAP target genes CTGF, CYR61, ANKRD1, and AMOTL2 (Figure 5C). Furthermore, KEGG pathway analysis showed significant enrichment of “Hippo Signaling pathway”-related genes in downregulated genes in VGLL4-expressing Ben-Men-1 cells (Figure 5D). In addition, VGLL4 overexpression led to a significant decrease in the growth of Ben-Men-1 cells compared to GFP-expressing cells (Figure 5E, Supplementary Figure 4B).

Taken together, these results confirm the direct link between VGLL4 overexpression and reduced levels of YAP activity observed in aggressive NF2 mutant meningiomas.

## Discussion

Around 50 percent of meningiomas reduce NF2 gene function, most commonly by loss of chromosome 22 (harboring NF2), but also inactivating mutations or gene fusions in the NF2 gene. NF2 has a myriad of tumor suppressor functions, including the regulation of the Ras-Raf-MEK-ERK and PI3K-AKT-mTOR-S6 pathways^27^; however, one of its pivotal functions involves the negative regulation of the oncogenes YAP1 and TAZ through the Hippo Signaling pathway. Loss of NF2 results in deregulation and activation of oncogenic YAP activity and increased levels of YAP activity have been observed in NF2 mutant over NF2 wild-type meningiomas.^7,8^ Oncogenic YAP activity has also been implicated in the pathobiology of other tumors harboring inactivating NF2 mutations.^28,29^ Recently, we have shown that the expression of a non-regulatable YAP1 variant (S127/397A-YAP1) induces very similar transcriptional changes compared to NF2 loss in human neural stem cells and that the expression of the same construct in Nestin-expressing cells in the meninges of *Cdkn2a* null mice induces the formation of meningioma-like tumors that resemble human NF2 mutant meningiomas by histomorphology and gene expression.^10^ This link between NF2 and YAP is further highlighted by the presence of YAP1 gene fusions in pediatric NF2 wild-type meningiomas, which cluster closely with NF2 mutant meningiomas based on DNA methylation and RNA Seq classifications.^10,9^

**Figure 6.**
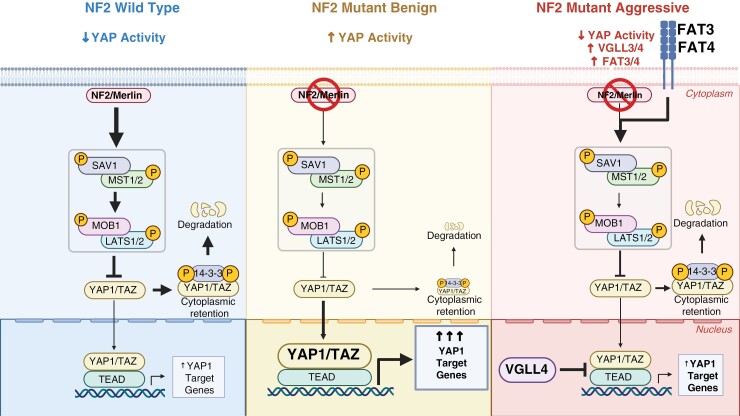
Schematic overview of YAP signaling in NF2 wild type as well as benign and aggressive NF2 mutant meningiomas. Figure created with biorender.com.

While inactivating NF2 mutations and loss of chromosome 22 are frequently the only recurring mutations in benign NF2 mutant meningiomas, higher-grade and aggressive NF2 mutant tumors generally harbor a more aberrant genome, suggesting that additional pathways are deregulated in these tumors. Our data suggests that aggressive NF2 mutant meningiomas downregulate at least a subset of the oncogenic YAP1 activity that is present in benign NF2 mutant meningiomas (Figure 6). We observed this effect across tumors of all higher-grade methylation subtypes (int-A, int-B, mal) when compared to tumors of the benign ben-1 subtype. Our data furthermore indicates that this downregulation of YAP activity can be achieved in several ways. Tumors of the mal subtype significantly upregulated the expression of the transcriptional co-factor VGLL4 (and to a lesser degree VGLL3). VGLL proteins, similar to YAP1, bind to TEAD transcription factors, thereby preventing the interaction between YAP1 and TEADs. VGLL1-3 each contains one Tondu (TDU) domain important for the interaction with TEADs, whereas VGLL4 contains two conserved TDUs that mediate the VGLL4-TEAD interaction. Except for their TDU domains, these proteins bear no sequence similarity, suggesting that VGLL1-3 and VGLL4 might have distinct molecular functions.^30,31^ Furthermore, while several studies have identified oncogenic functions of VGLL1-3 in different cancer types,^23,32–35^ VGLL4 has so far only been described as a tumor suppressor, and reduced VGLL4 expression levels have been detected in several cancers.^36–39^ However, the exact functions of VGLL4 in tumorigenesis or progression remain unclear.

Tumors of all aggressive subtypes, while more pronounced in tumors of the int-A and int-B subtypes, also overexpressed FAT4. Mal subtype tumors also upregulated the expression of FAT3. In Drosophila, FAT can activate the Hippo pathway independently of NF2, and, while the exact mechanism is not conserved in mammals, mammalian FAT family proteins (FAT1-4) have been implicated in the regulation of YAP1.^40–43^ Loss or functional inactivation of FAT proteins has also been observed in benign NF2 wild type spinal meningiomas, suggesting tumor suppressive activity of FAT proteins in benign meningioma.^44^

In vitro data with benign NF2 mutant Ben-Men-1 meningioma cells and HEK293 cells showed that overexpression of VGLL4 results in the significant suppression of YAP1 activity, confirming the direct link between overexpression of VGLL4 and reduced YAP activity. Functional inhibition or loss of expression of YAP1 has been observed in several cancers, such as Estrogen receptor (ER)-positive breast cancer, neuroendocrine small-cell lung cancer (SCLC), or pulmonary large-cell neuroendocrine carcinoma.^45–48^ YAP1 was originally identified as a tumor suppressor due to its role in activating p73-dependent apoptosis^49^ and has been shown to suppress ER expression in ER+ breast cancer as well as suppress metastasis in SCLC.^46,48^ However, it is unclear if and how decreased YAP activity benefits the tumor cells in aggressive NF2 mutant meningiomas. The reason for the inhibition of YAP activity in these tumors remains unclear and warrants further investigation.

It is currently unknown if benign and aggressive NF2 mutant meningiomas equally rely on YAP1-TEAD signaling for their growth and/or survival. YAP1 is a transcriptional coactivator that does not bind DNA itself, but instead relies on the interaction with other transcription factors (mostly TEAD1-4). Several pharmacological inhibitors of YAP signaling are currently being developed, most of them inhibiting the interaction of YAP1 with TEAD transcription factors, and their efficacy is currently being assessed in phase 1 clinical trials of several YAP1-activated tumor types (such as NF2 mutant mesothelioma).^13,50^ It remains unclear if YAP1-TEAD inhibitors are equally effective against both benign (that seem to largely rely on YAP signaling) and aggressive NF2 mutant meningiomas (that harbor additional mutations and likely concurrently activate additional mitogenic pathways), especially since aggressive tumors show decreased baseline levels of YAP activity. To assess this, the efficacy of YAP1-TEAD inhibitors needs to be evaluated on a larger number of cell lines and/or human tumor samples, with substratification based on molecular grade and subtype. For example, do aggressive meningiomas that downregulate YAP activity via FAT3/4 overexpression respond in a similar fashion compared to tumors expressing VGLL4? Additionally, it remains to be shown if a similar downregulation of YAP signaling is also present in aggressive types of other CNS or non-CNS tumors that harbor NF2 mutations, such as NF2 mutant spinal ependymomas or mesothelioma, and might be a general phenomenon.

In summary, our study suggests that aggressive NF2 mutant tumors downregulate oncogenic YAP activity, by both upregulating the expression of the YAP1 antagonist VGLL4 and by upregulating the upstream regulators FAT3/4. Our findings may have important implications for the efficacy of therapeutic approaches aimed at the YAP1-TEAD complex in benign versus aggressive NF2 mutant meningiomas.

## Supplementary Material

vdae148_suppl_Supplementary_Data

vdae148_suppl_Supplementary_Table_S1

vdae148_suppl_Supplementary_Table_S2

## Data Availability

The data that support the findings of this study are included with the manuscript and supplemental data files and are also available from the corresponding author upon reasonable request.
