# Supplementary material for: Aggressive high-grade NF2 mutant meningiomas downregulate oncogenic YAP signaling via the upregulation of VGLL4 and FAT3/4
Source: Neurooncol Adv. 2024 Aug 24;6(1):vdae148. doi: 10.1093/noajnl/vdae148 (PMC11459063; doi:10.1093/noajnl/vdae148)
Supplement: vdae148_suppl_Supplementary_Data [file vdae148_suppl_supplementary_data.pdf]

Aggressive high-grade NF2 mutant meningiomas downregulate oncogenic YAP signaling via the upregulation of VGLL4 and FAT3/4.

Abigail G Parrish[1†], Sonali Arora[1†], H. Nayanga Thirimanne[1], Dmytro Rudoy[1], Sebastian Schmid[1], Philipp Sievers[2,3], Felix Sahm[2,3,4], Eric C Holland[1,5], Frank Szulzewsky[1,*]

Supplementary Information

Table of Contents:

Supplementary Figures S1-S4
Supplementary Figure and Table Legends

Suppl. Figure S1

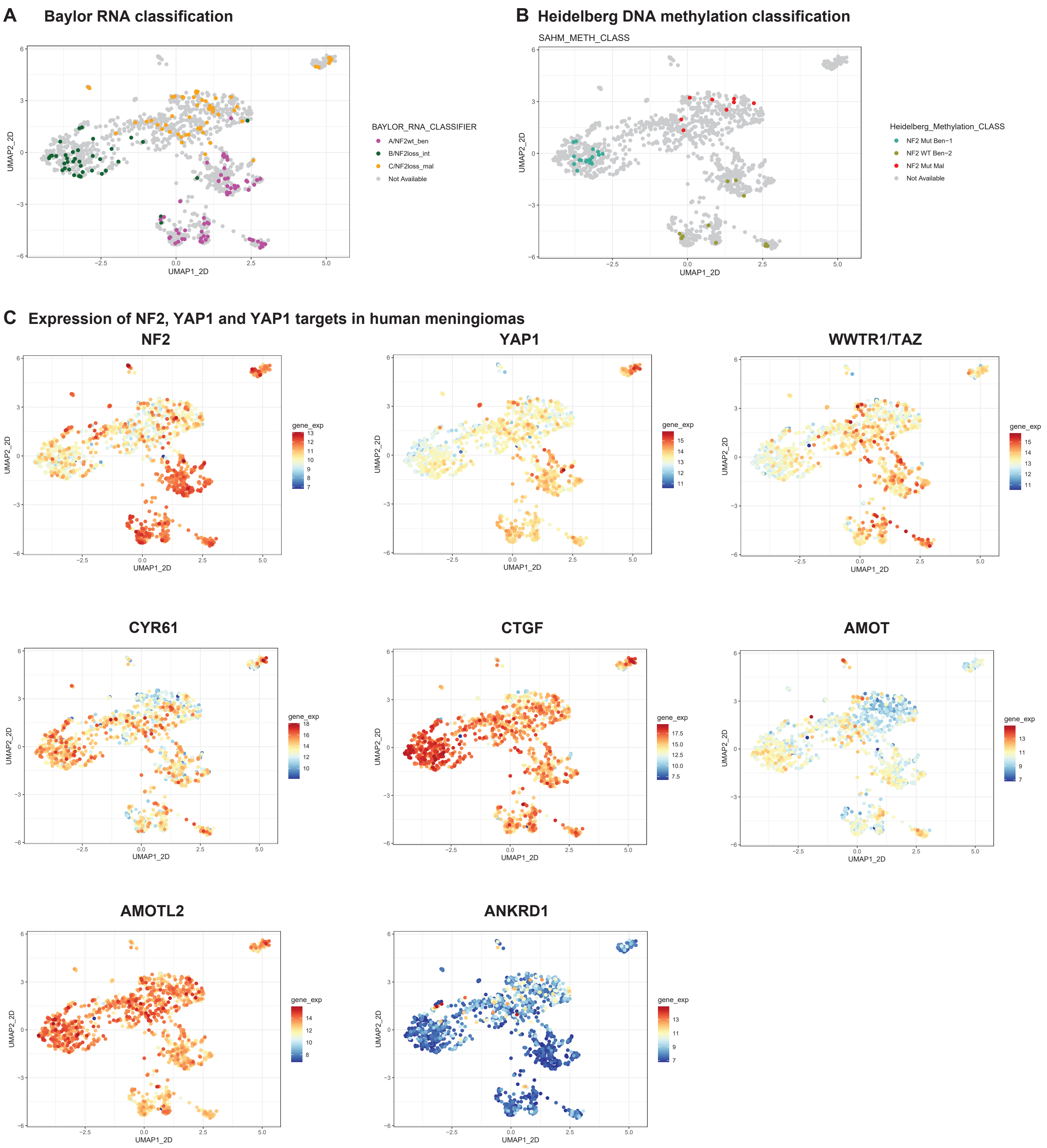

Suppl. Figure S2

A CNS WHO grade - All NF2 mutant meningiomas (cluster A+B)

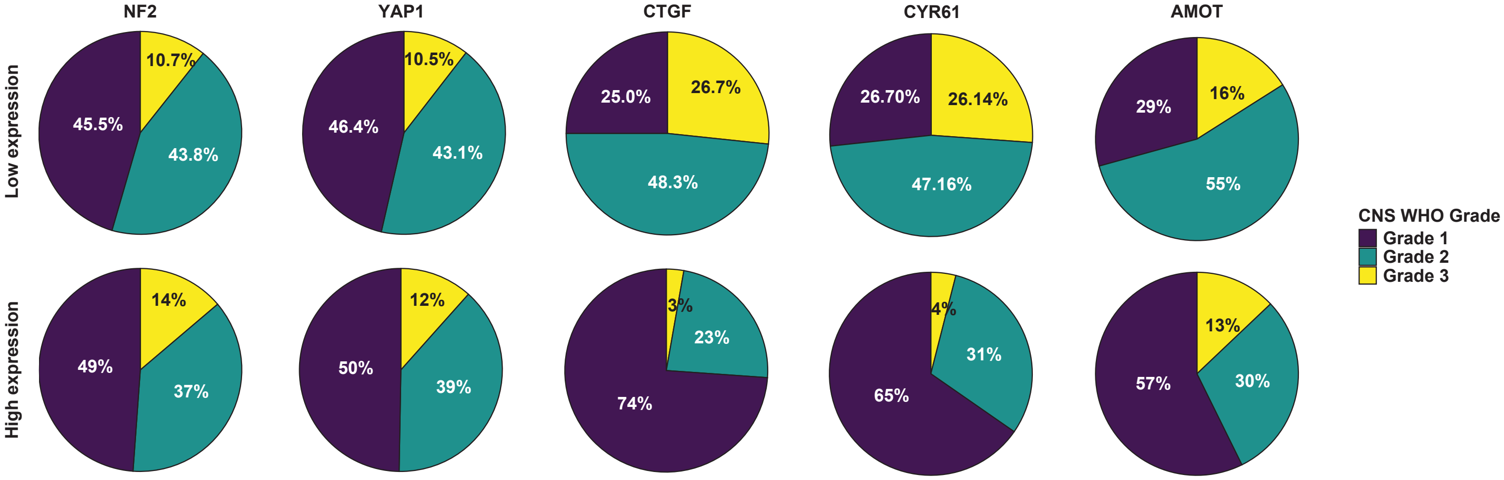

Suppl. Figure S3A

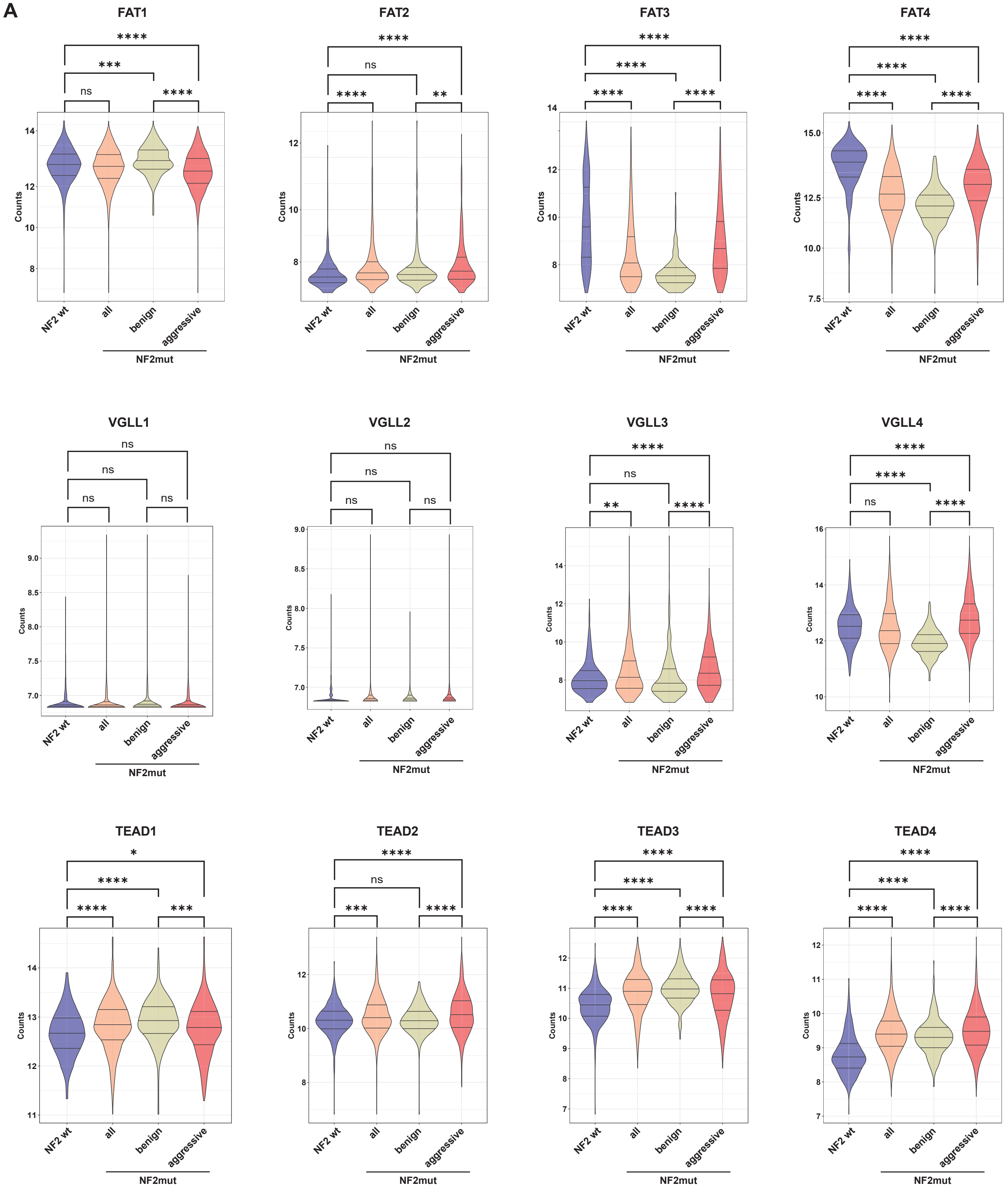

Suppl. Figure S3A (continued)

A (continued)

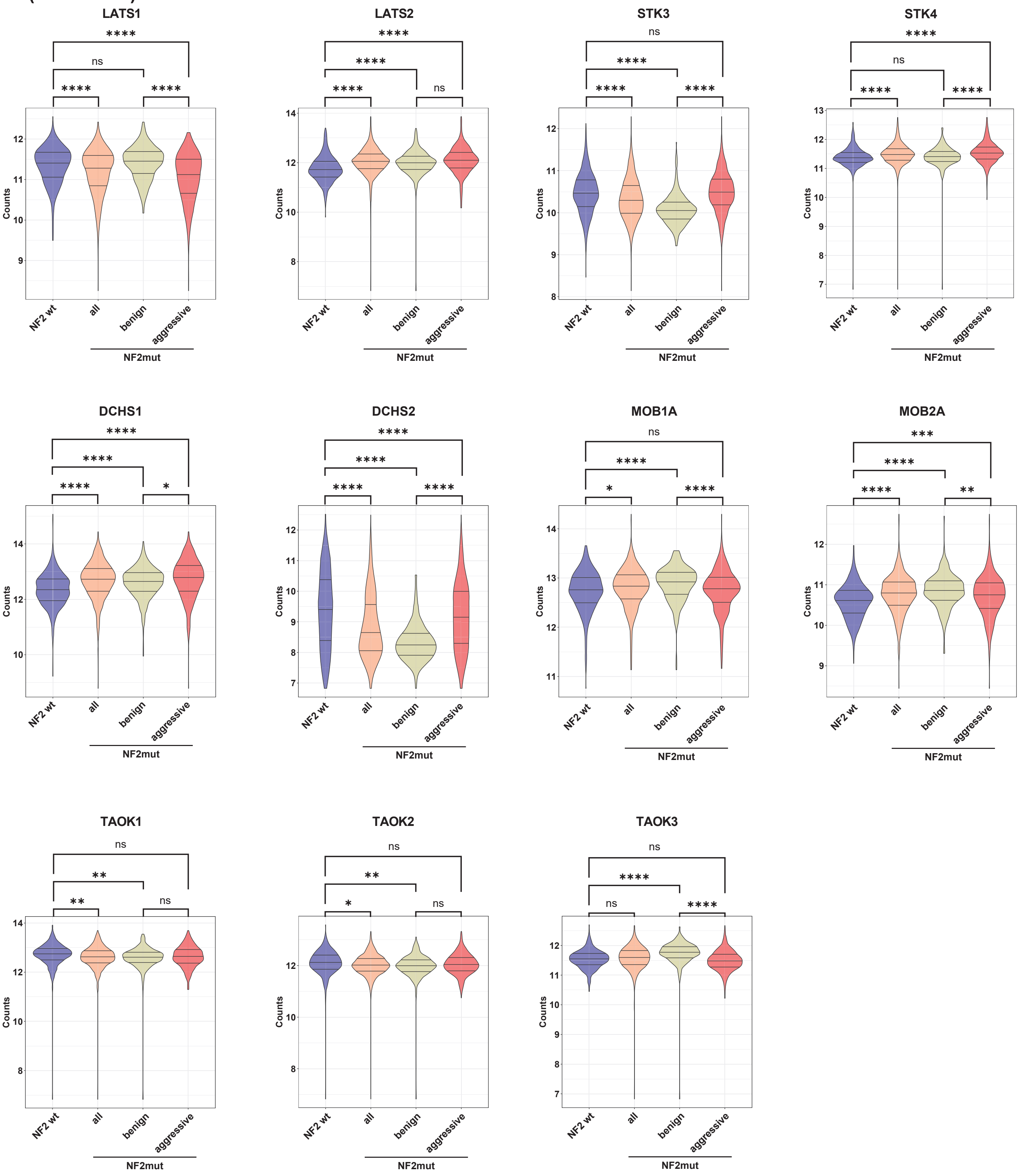

Suppl. Figure S3A (continued)-C

A (continued)

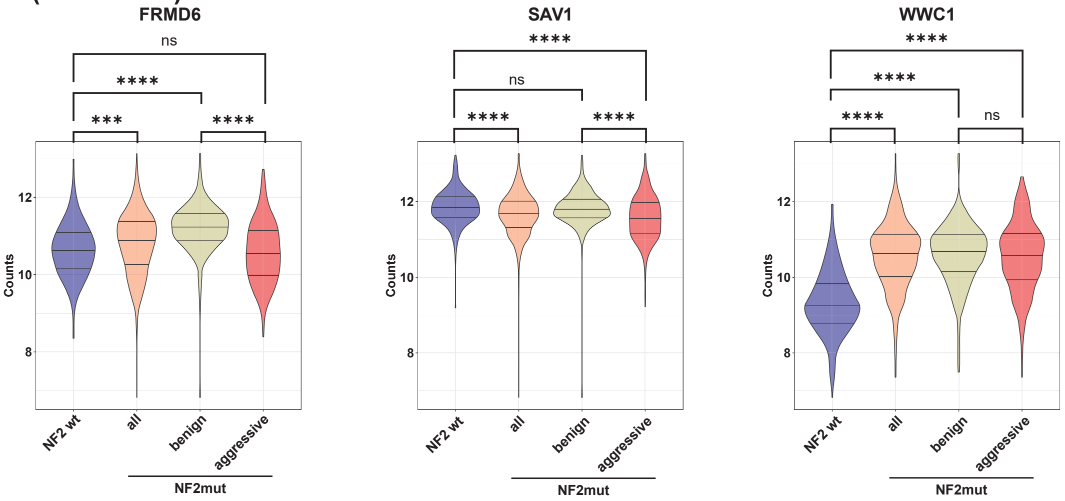

B

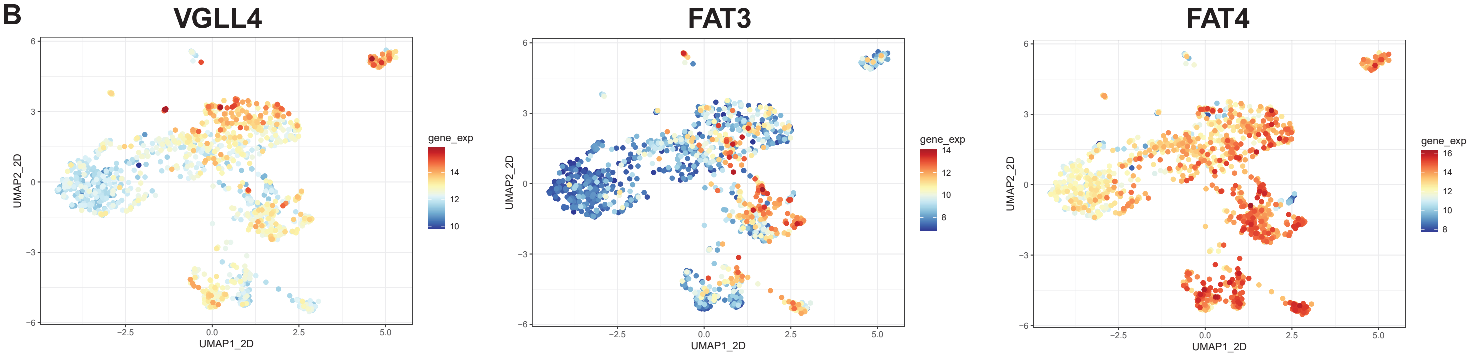

C Time to recurrence - all NF2 mutant meningiomas (cluster A+B)

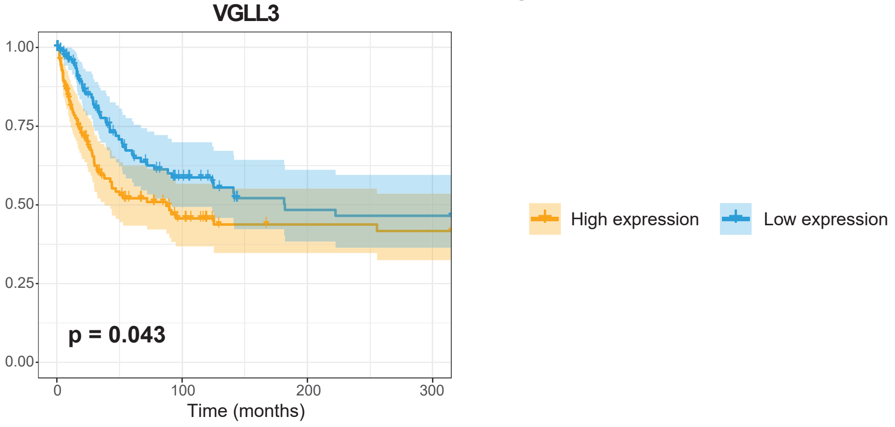

Suppl. Figure S3D

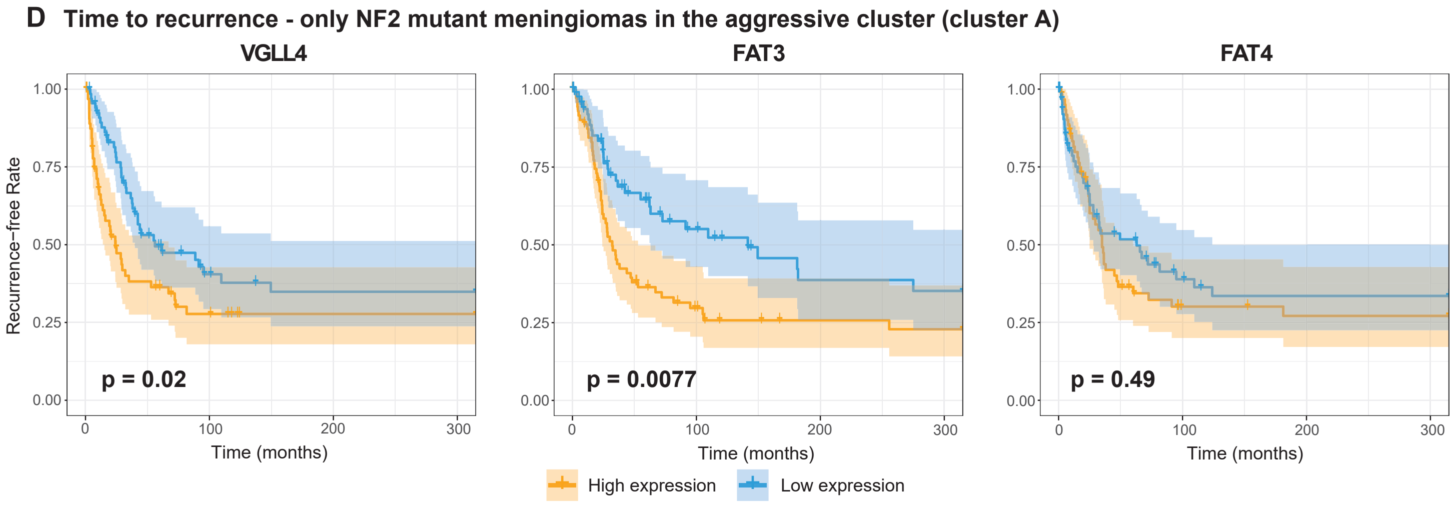

Suppl. Figure S4

A

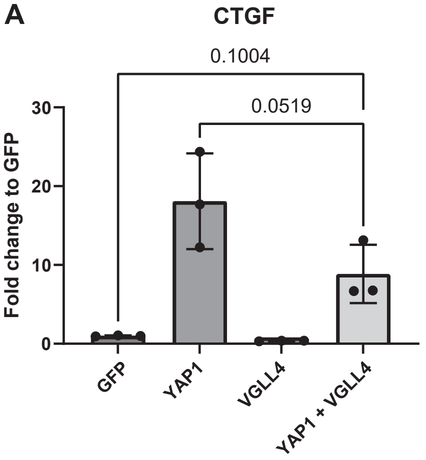

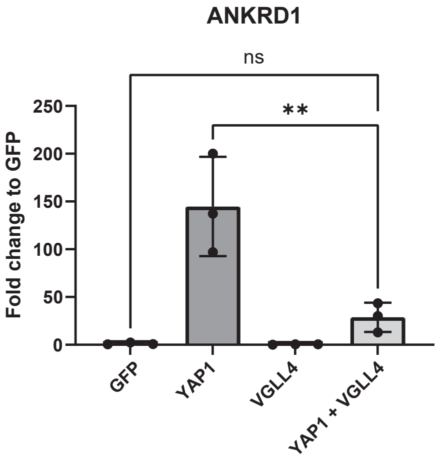

B

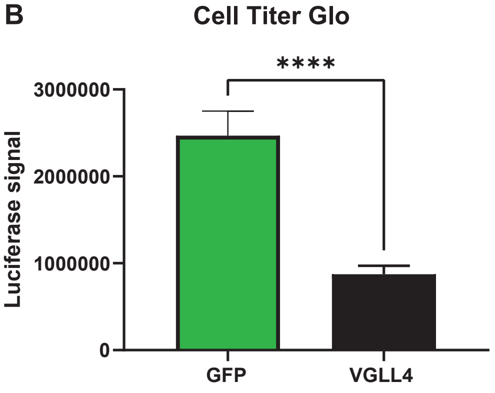

Suppl. Figure and Table legends:

Suppl. Figure S1: Aggressive NF2 mutant meningiomas display decreased levels of YAP activity. A-C) Reference UMAPs showing clustering of human meningiomas based on bulk RNA-Seq data. Samples are colored by Baylor RNA classification status (A), Heidelberg methylation classifier status (B), or gene expression of several Hippo Pathway members and YAP1 target genes (C). Statistical analysis was done with One-way ANOVA (B). (***) $P \leq 0.001$; (****) $P \leq 0.0001$.

Suppl. Figure S2: Low expression of YAP1 target genes is associated with shorter time to recurrence in NF2 mutant meningioma. A) WHO grades of tumors harboring low or high expression of either NF2 and YAP1 or the YAP targets CTGF, CYR61, and AMOT.

Suppl. Figure S3: Aggressive NF2 mutant meningiomas upregulate the expression of VGLL4, FAT3, and FAT4. A) Expression (VST counts) of Hippo Pathway genes in bulk RNA-Seq data of NF2 wild type, as well as NF2 mutant (benign and aggressive) human meningiomas. B) Reference UMAPs showing clustering of human meningiomas based on bulk RNA-Seq data. Samples are colored by gene expression of VGLL4, FAT3, and FAT4. C) Time to recurrence of NF2 mutant human meningiomas tumors harboring low or high expression of VGLL3. D) Time to recurrence of NF2 mutant human meningiomas (aggressive subtype tumors only) harboring low or high expression of VGLL4, FAT3, or FAT4. Statistical analysis was done with One-way ANOVA (A) or Log-rank (Mantel-Cox) test (C, D). (*) $P \leq 0.05$; (**) $P \leq 0.01$; (***) $P \leq 0.001$; (****) $P \leq 0.0001$.

Suppl. Figure S4: Expression of VGLL4 leads to the suppression of YAP target genes in vitro. A) Expression of the YAP1 target genes CTGF and ANKRD1 in HEK cells upon transient transfection of either GFP, 2SA-YAP1, VGLL4, or 2SA-YAP1 + VGLL4. B) Cell titer Glo data of Ben-Men-1 cells expressing either VGLL4 or GFP after 5.5 days of culturing in 96 well plates. Statistical analysis was done with One-way ANOVA (A) or t test (B). (**) $P \leq 0.01$.

Supplementary Table S1: A) Sample info of the 26 single-cell RNA-Seq samples of human meningiomas. B) List of marker genes used to filter out immune cells from single-cell RNA-Seq data.
Supplementary Table S2: A) List of primers used/ B) List of plasmids used.
